# BMI1 reduces ATR activation and signalling caused by hydroxyurea

**DOI:** 10.18632/oncotarget.21111

**Published:** 2017-09-20

**Authors:** Xiaozeng Lin, Fengxiang Wei, Peter Whyte, Damu Tang

**Affiliations:** ^1^ Division of Nephrology, Department of Medicine, McMaster University, Hamilton, Ontario, Canada; ^2^ Father Sean O’Sullivan Research Institute, Hamilton, Ontario, Canada; ^3^ The Hamilton Center for Kidney Research, St. Joseph's Hospital, Hamilton, Ontario, Canada; ^4^ The Genetics Laboratory, Longgang District Maternity and Child Healthcare Hospital, Longgang District, Shenzhen, Guangdong, P.R. China; ^5^ Department of Pathology and Molecular Medicine, McMaster University, Hamilton, Ontario, Canada

**Keywords:** BMI1, DNA damage response, ATR, CHK1, S checkpoint activation

## Abstract

BMI1 facilitates DNA damage response (DDR) induced by double strand DNA breaks; however, it remains unknown whether BMI1 functions in single strand DNA (ssDNA) lesions-initiated DDR. We report here that BMI1 reduces hydroxyurea-elicited ATR activation, thereby reducing the S-phase checkpoints. Hydroxyurea induces ssDNA lesions, which activate ATR through binding TOPBP1 as evidenced by phosphorylation of ATR at threonine 1989 (ATRpT1989). ATR subsequently phosphorylates H2AX at serine 139 (γH2AX) and CHK1 at serine 345 (CHK1pS345), leading to phosphorylation of CDK1 at tyrosine 15 (CDK1pY15) and S-phase arrest. BMI1 overexpression reduced γH2AX, CHK1pS345, CDK1pY15, S-phase arrest, and ATR activation in HU-treated MCF7 and DU145 cells, whereas BMI1 knockdown enhanced these events. BMI1 contains a ring finger, helix-turn, proline/serine domain and two nuclear localization signals (NLS). Individual deletion of these domains did not abolish BMI1-derived reductions of CHK1pS345 in MCF7 cells following HU exposure, suggesting that these structural features are not essential for BMI1 to attenuate ATR-mediated CHK1pS345. BMI1 interacts with both TOPBP1 and ATR. Furthermore, all of our BMI1 mutants associate with endogenous TOPBP1. It has previously been established that association of TOPBP1 and ATR is required for ATR activation. Thus, our results suggest that BMI1 decreases ATR activation through a mechanism that involves binding to TOPBP1 and/or ATR.

## INTRODUCTION

BMI1 is a polycomb group (PcG) protein of the polycomb repressive complex 1 (PRC1) [1], and is required for formation of E3 ubiquitin ligase activity of PRC1 via binding to the catalytic subunit RING2 [2–5]. The E3 ubiquitin ligase activity underlies PRC1-mediated suppression of gene expression. For example, BMI1 represses the *INK4A/ARF* and *E4F1* loci [6–9]. The *INK4A/ARF* locus encodes two tumor suppressors, p16^INK4A^ and p19^ARF^/p14^ARF^, via alternative splicing and using differential promoters [10, 11]. E4F1 inhibits cell proliferation, in part, through promoting p53 and CHK1 functions [12–14]. Suppression of these loci contributes to BMI1-derived maintenance of the self-renewal of hematopoietic and neural stem cells [8, 15, 16]. In some cases, inhibition of INK4A and ARF-mediated tumor suppression is critical for tumorigenesis [10, 11] and upregulation of BMI1 occurs in numerous cancer types including non-small cell lung cancer [17], colon cancer [18], breast cancer [19], and nasopharyngeal carcinoma [20]. BMI1 overexpression is able to transform lymphocytes [21, 22] and its upregulation in lymphomas associates with poor prognosis [23–25]. Also, expression of BMI1 can synergize with c-Myc in transgenic mouse models for leukemogenesis [26, 27].

In addition to inhibition of the pRB and p53 tumor suppressors through repression of the *INK4A/ARF* locus [10, 11], BMI1 is involved in DNA damage response (DDR) [28–31]. DDR is essential in maintenance of genomic integrity and accurate passage of genetic materials to the daughter cells [32]. Compromising DDR leads to genomic instability, a hallmark of cancer [33, 34] and a major cause of tumorigenesis [35–37]. Enhancing repair of DDR lesions contributes to therapy resistance in cancer [38, 39]. DDR is initiated by a variety of DNA lesions [40] through activation of three apical PI3 kinase-related kinases (PIKKs) ATM, ATR, and DNA-PK [41, 42]. PIKKs coordinate DDR via checkpoint activation to prevent cell cycle progression and preparation for DNA lesion repair [32, 43]. Double strand DNA breaks (DSBs) activate ATM, leading to phosphorylation of downstream targets, including CHK2 and γH2AX [41, 42]. CHK2 activation subsequently results in G2/M arrest [32, 43] and the formation of γH2AX nuclear foci around DSBs initiates DSB repair [44, 45]. As part of the repair process, BMI1 rapidly associates with DSBs, ubiquitinates γH2AX, and contributes to homologous recombination (HR)-facilitated DSB repair [28–30]. Additionally, BMI1 also compromises DSB-induced checkpoint activation by reducing ATM activation [31].

Another major arm of DDR is initiated by single-strand DNA (ssDNA) lesions, which are typically produced by stalled replication forks. These lesions are first coated with replication protein A (RPA). RPA-ssDNA independently recruits the ATR-ATRIP complex and TOPBP1, where TOPBP1 activates ATR through a physical association. ATR subsequently phosphorylates and activates CHK1, leading to S-phase arrest [46, 47]. In view of these similarities between ATM and ATR activation, we have examined whether BMI1 also decreases ssDNA-initiated ATR activation.

Hydroxyurea (HU) is a potent DNA synthesis inhibitor [48], and causes stalled replication forks through depletion of the dNTP pool, leading to accumulation of ssDNA and activation of the ATR-dependent S-phase checkpoints [49]. We report here that BMI1 delays S-phase checkpoint activation induced by HU. In MCF7 cells treated with HU, BMI1 overexpression reduced ATR activation, phosphorylation of CHK1, and S-phase arrest, while BMI1 knockdown had the opposite effect. BMI1 interacted with TOPBP1 and ATR in co-immunoprecipitation experiments suggesting a possible mechanism.

## RESULTS

### BMI1 delays HU-induced activation of the S-phase checkpoints

**Figure 1:**
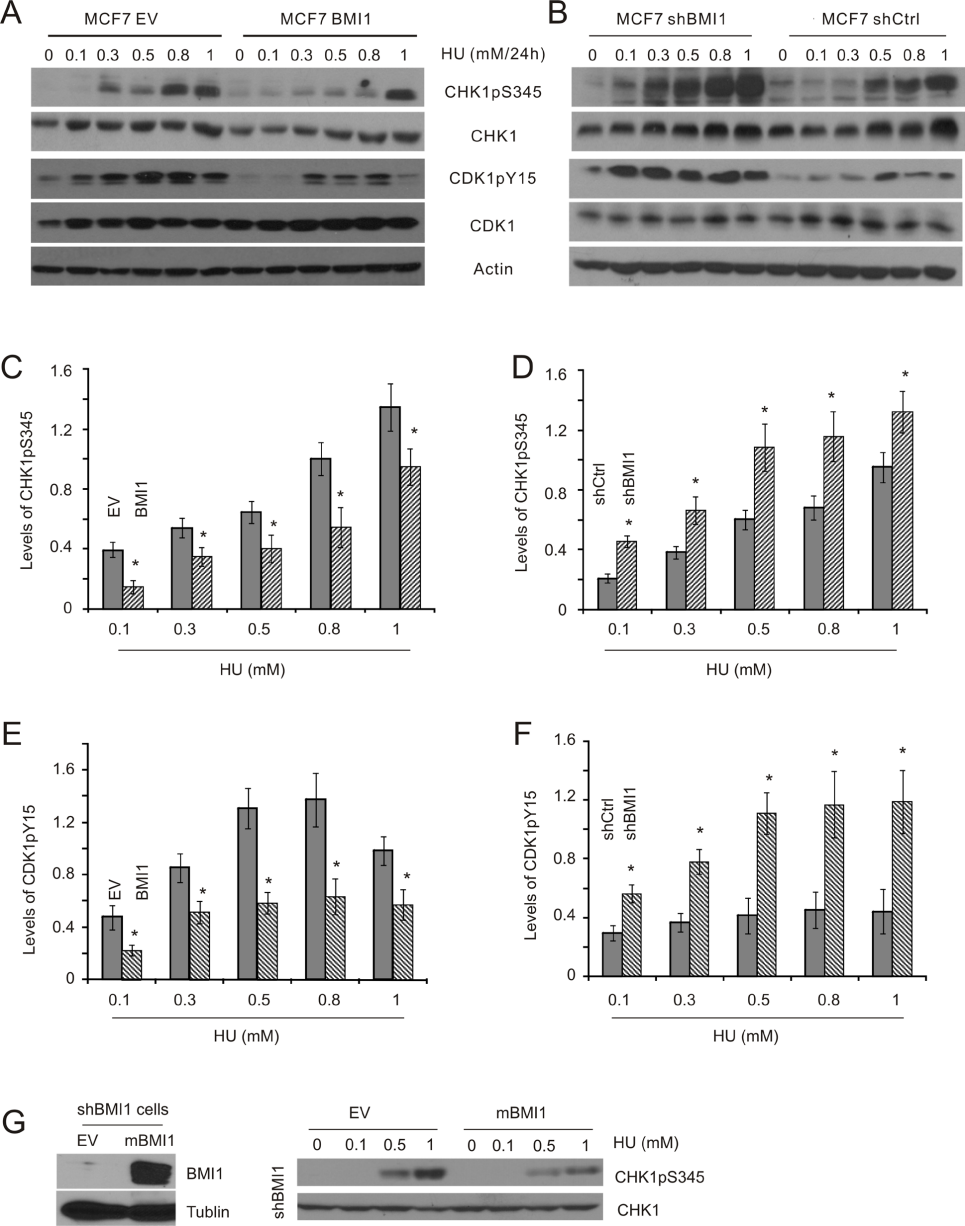
BMI1 decreases HU-induced CHK1 activation in MCF7 cells MCF7 EV (empty vector), BMI1, shCtrl (Ctrl: control), and shBMI1 stable cell lines were established (see Supplementary Figure 1). These cell lines were treated with HU at the indicated doses for 24 hours, followed by Western blot examination for CHK1 phosphorylation at S345 (CHK1pS345), CHK1, CDK1 phosphorylation at Y15 (CDK1pY15), CDK1, and actin. Experiments were carried out three times; typical results from a single repeat are shown (**A, B**); means ± S.E (standard error) were graphed (**C–F**). *p < 0.05 in comparison to the respective control (EV and shCtrl) cells (two-tailed Student's *t*-test). (**G**) MCF7 shBMI1 cells were stably transfected with EV or mouse BMI1 (mBMI1) (left panel) [31], followed by the examination of HU-induced CHK1pS345. The experiments were repeated once; typical images from a single repeat are included.

BMI1 has been reported to enhance HR-mediated DSB repair [28–30], and reduce DSB-initiated G2/M checkpoints caused by etoposide [31]. To investigate whether BMI1 is involved in ssDNA-stimulated DDR, we have constructed MCF7 breast cancer and DU145 prostate cancer cell lines in which BMI1 was either stably overexpressed or knocked-down (Supplementary Figure 1). Using these lines, we examined the impact of BMI1 expression levels on HU-induced S-phase checkpoints. Previous studies have demonstrated that HU causes stalled replication forks as a result of depletion of cellular dNTP pools, leading to activation of the S-phase checkpoints in MCF7 and DU145 cells [50, 51] through CHK1 activation. CHK1 contributes to CDK1 inactivation via sustaining CDK1 phosphorylation at tyrosine 15 (Y15) [52], an event that prevents mitotic entry [53]. Accordingly, treatment of the cells with HU resulted in a dose-dependent stimulation of CHK1 phosphorylation at serine 345 (CHK1pS345), indicative of CHK1 activation, and CDK1pY15 in both MCF7 (Figure 1A, 1C, and 1E) and DU145 cells (Supplementary Figure 2). Both events were substantially reduced in MCF7 BMI1 (Figure 1A, 1C, and 1E). Although there was an unexpectedly high level of CDK1pY15 in DU145 BMI1 cells treated with 0.5mM HU, it is apparent that DU145 BMI1 cells displayed a lower kinetics of CDK1pY15 in response to HU exposure compared to DU145 EV cells (Supplementary Figure 2). Collectively, this evidence suggests that enforced BMI1 expression in both MCF7 and DU145 cells results in reductions in CHK1 activation and CDK1 inactivation following HU treatment. Conversely, knockdown of BMI1 elevated HU-induced CHK1pS345 and CDK1pY15 in MCF7 cells (Figure 1B, 1D, and 1F). Furthermore, we have stably re-expressed murine BMI1 into MCF7 shBMI1 cells [31] and confirmed its expression (Figure 1G); re-expression of murine BMI1 reversed the elevation of CHK1pS345 in MCF7 shBMI1 cells treated with HU (compare the CHK1pS345 profile in Figure 1B to that in Figure 1G), indicating that the increase in CHK1pS345 following BMI1 knockdown in MCF7 cells (Figure 1B) was not caused by potential off-target effects. Examination of HU-induced checkpoint activation has been commonly performed by treating cells for 24 hours at concentrations of 1mM or less [50, 51]. This condition does not result in substantial collapse of the DNA replication forks, as cells renewed proliferation upon releasing from 1mM HU-24 hour treatment with minimal adverse effects in comparison to mock-treated cells (Supplementary Figure 3).

**Figure 2:**
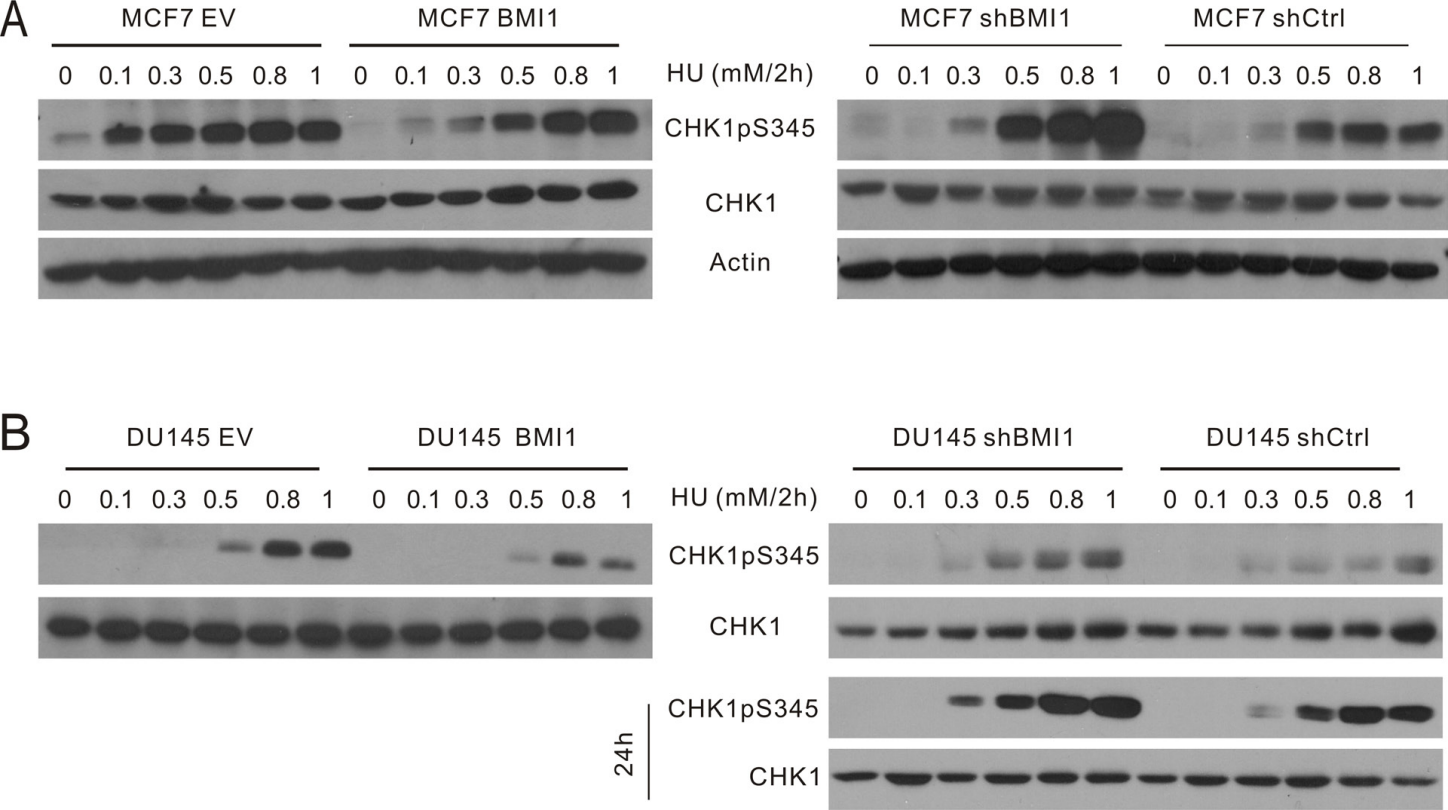
BMI1 reduces the early onset of CHK1pS345 caused by HU The MCF7 (**A**) and DU145-based (**B**) EV, BMI1, shCtrl, and shBMI1 cells were treated with the indicated doses of HU for 2 hours, followed by determination of CHK1pS345, CHK1, and actin by Western blot. For DU145 shCtrl and shBMI1 cells, the indicated treatments were also continued for 24 hours (bottom right panel). Experiments were repeated once; typical images from a single repeat are shown.

To further evaluate conditions involving 1mM HU or less for 24 hours, we determined the kinetics of CHK1 activation in MCF7 cells following HU treatment. HU elicited early (1–8 h) and late phases (14–24 h) of CHK1 activation (Supplementary Figure 4). As cell cycle arrest, a major consequence of checkpoint activation, is commonly examined at 24 hour following treatment [50, 51], we focused this study on the late phase of CHK1 activation and DDR induced by HU at doses ≤ 1 mM. Nevertheless, because HU also induces an early onset of CHK1pS345 (Supplementary Figure 4), we have determined whether BMI1 affected CHK1pS345 in this early phase. It was clear that overexpression of BMI1 in both MCF7 and DU145 cells robustly reduced the dose-dependent kinetics of CHK1pS345 during a 2-hour HU treatment (Figure 2A, 2B, left panels), whereas knockdown of BMI1 in both lines elevated this event (Figure 2A, 2B, right panels). Furthermore, knockdown of BMI1 in DU145 cells also reduced HU-induced late-phase CHK1pS345 in compared to DU145 shCtrl cells (Figure 2B, bottom right panel). Similar results were also obtained in MCF7 shBMI1 cells (Figure 1B, 1D). BMI1 overexpression in MCF7 and DU145 cells reduced HU-induced CHK1pS345 at 24-hour HU treatment (Figure 1A, 1C; Supplementary Figure 2). Nonetheless, the effects of modulation of BMI1 on CHK1pS345 in cells treated with high doses of HU, particularly 1mM HU appeared less robust compared to cells treated with low doses of HU in response to either late (24 h) or early phase (2h) treatment (Figures 1 and 2). These observations indicate that BMI1 attenuates CHK1 activation and, collectively, the above experiments provide a comprehensive set of data demonstrating a role of BMI1 in reducing CHK1 activation following HU treatment.

### BMI1 reduces ATR activation caused by HU

**Figure 3:**
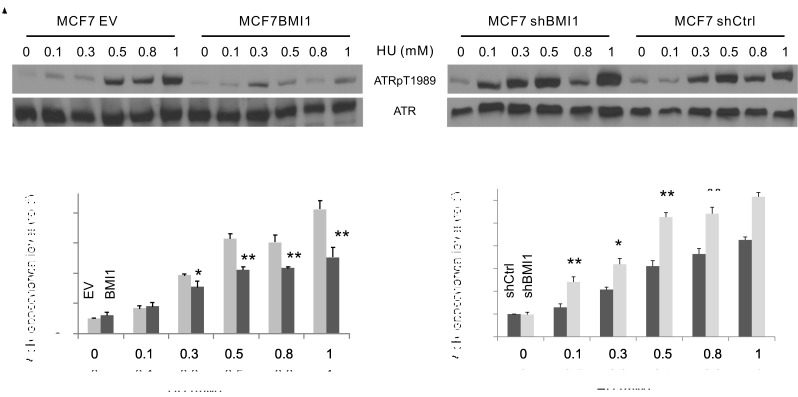
BMI1 reduces ATR activation Examination of phosphorylation of ATR at T1989 (ATRpT1989) in the indicated MCF7 cell lines. Experiments were repeated three times; typical images from a single repeat are shown (**A**). (**B, C**) ATR phosphorylation was normalized to the respective ATR and expressed as fold changes to the normalized ATR phosphorylation levels in the respective (EV and shCtrl) control (untreated) cells. Means ± SD (standard derivation) were graphed; *p < 0.05 and **p < 0.01 by 2-tailed Student's *t*-test in comparison to the respective controls.

The above observations led us to hypothesise that BMI1 reduces ATR activation. ATR activation depends on autophosphorylation at threonine 1989 (ATRpT1989) [54]. In this regard, BMI1 overexpression reduced ATRpT1989 in HU-treated MCF7 cells (Figure 3A, left panel; Figure 3B), whereas BMI1 knockdown enhanced the event (Figure 3A, right panel; Figure 3C). These observations support the concept that BMI1 attenuates HU-induced ATR activation.

**Figure 4:**
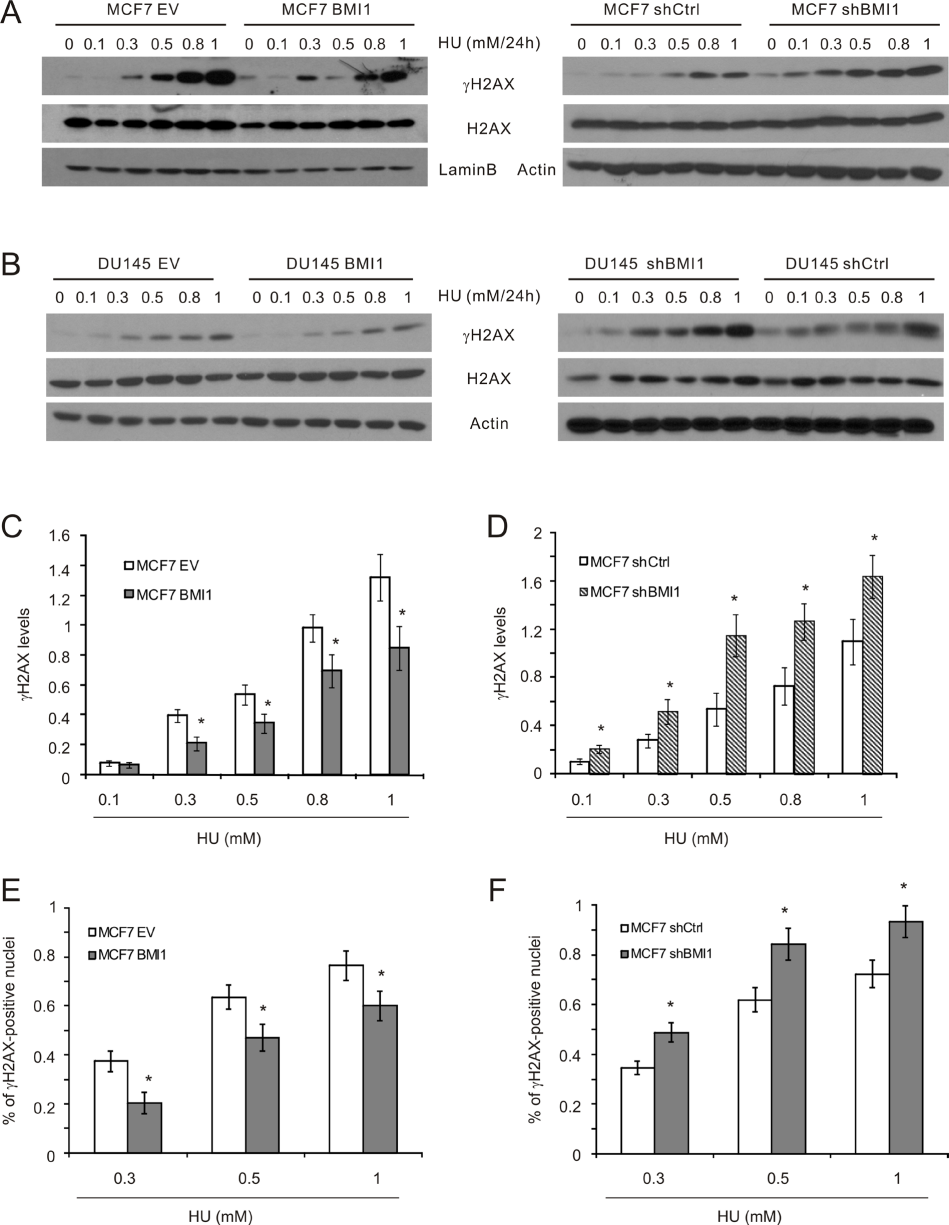
BMI1 attenuates γH2AX in cells treated with HU (**A, B**) The indicated MCF7 and DU145 cell lines were exposed to HU at the indicated doses for 24 hours. The indicated events were examined by Western blot. Experiments were repeated three times. Typical results from a single repeat were included (A, B). γH2AX results for MCF7 EV, BMI1 (**C**), shCtrl, and shBMI1 (**D**) cells were quantified. Means ± S.E were graphed. *p < 0.05 in comparison to the respective control cells (two-tailed Student's *t*-test). (**E, F**) MCF7 EV, BMI1 (E), shCtrl, and shBMI1 (F) cells were treated with PBS or HU for 24 hours. IF staining for γH2AX was performed. Typical images are presented in Supplementary Figure 5. Experiments were repeated three times; γH2AX-positive nuclei were quantified; mean percentages ± S.E are graphed. *p < 0.05 in comparison to the respective control cells (two-tailed Student's *t*-test).

This notion is further supported by the effects of BMI1 on γH2AX, a target of ATR, in MCF7 cells [47]. Western blot analysis revealed a reduction of γH2AX in MCF7 BMI1 cells in comparison to MCF7 EV cells in response to HU treatment (Figure 4A, 4C). Similar results were also obtained in DU145 BMI1 cells (Figure 4B). Conversely, knockdown of BMI1 in both MCF7 and DU145 cells elevated HU-induced γH2AX levels (Figure 4A, 4B, 4D). Additionally, formation of γH2AX nuclear foci, an apical and essential event for DSB repair [55, 56], was respectively reduced and enhanced in MCF7 BMI1 and shBMI1 cells in comparison to the respective control cells following HU exposure (Figure 4E, 4F; Supplementary Figure 5).

### BMI1 attenuates HU-induced S phase arrest

ATR is required for S-phase arrest in response to HU-induced DNA damage [50, 51]. To determine whether BMI1 affected this process, HU treated cells were examined for S phase cell cycle arrest. In MCF7 EV cells, HU treatment resulted in the expected dose-dependent accumulation in S phase that is indicative of S phase arrest (Table 1; Supplementary Figure 6). In the MCF7 BMI1 cells, the accumulation of S phase cells was significantly reduced (Table 1; Supplementary Figure 6); whereas, BMI1 knockdown increased the proportion of S phase cells (Table 2; Supplementary Figure 6) suggesting that BMI1 reduces HU-induced S phase arrest.

**Table 1:** BMI1 reduces HU-induced S arrest in MCF7 cells

	CTRL	0.1 mM	0.3 mM	0.5 mM	0.8 mM	1.0 mM
	EV BMI1	EV BMI1	EV BMI1	EV BMI1	EV BMI1	EV BMI1
G1	64.1 ± 1.8 65.3 ± 2.5	59.7± 0.1 58.7 ± 1.5	57.4 ± 4.2 58.9 ± 3.1	40.0 ± 2.2 52.9 ± 1.5*	33.6 ± 2.9 51.4 ± 1.3*	23.8 ± 1.1 45.1 ± 0.6*
S	15.8 ± 0.5 14.2 ± 1.3	24.5 ± 0.8 18.6 ± 0.4*	31.0 ± 1.8 19.3 ± 0.9*	51.6 ± 2.2 28.7 ± 0.6*	59.7 ± 3.9 33.7 ± 2.2*	68.8 ± 3.1 36.9 ± 0.1*
G2/M	20.1 ± 2.0 20.5 ± 1.9	15.8 ± 0.8 22.7 ± 1.2*	11.6 ± 3.5 21.7 ± 2.2	11.4 ± 1.5 18.4 ± 1.1*	6.7 ± 1.4 14.9 ± 0.9*	7.4 ± 2.1 18.0 ± 0.5*

Note: Cell cycle distributions were derived from three independent experiments and were presented as means ± S.E.; Ctrl: control; * *p* < 0.05 (in comparison to the respective cell cycle distributions of EV cells)

**Table 2:** Knockdown of BMI1 enhances HU-induced S arrest in MCF7 cells

	CTRL	0.1 mM	0.3 mM	0.5 mM	0.8 mM	1.0 mM
	shCtrl shBMI1	shCtrl shBMI1	shCtrl shBMI1	shCtrl shBMI1	shCtrl shBMI1	shCtrl shBMI1
G1	64.6 ± 2.3 67.8 ± 0.7	59.0 ± 0.8 56.1 ± 3.0	62.3 ± 1.6 26.3 ± 3.7*	44.9 ± 4.6 20.1 ± 2.5*	29.5 ± 4.4 10.0 ± 0.2*	23.3 ± 0.6 9.8 ± 1.9*
S	19.5 ± 3.4 13.5 ± 0.8	24.4 ± 0.8 26.6 ± 1.4	26.6 ± 0.5 48.2 ± 3.1*	43.0 ± 5.6 66.7 ± 1.2*	64.2 ± 4.2 78.1 ± 1.0*	71.2 ± 1.2 80.9 ± 1.4*
G2/M	17.0 ± 1.1 18.7 ± 1.5	16.6 ± 0.8 17.2 ± 2.4*	11.1 ± 1.8 25.5 ± 0.7	12.1 ± 2.0 13.3 ± 2.1*	6.3 ± 1.0 12.0 ± 1.2	5.5 ± 0.9 9.3 ± 0.5*

Note: Cell cycle distributions were derived from three independent experiments and were presented as means ± S.E.; Ctrl: control; * *p* < 0.05 (in comparison to the respective cell cycle distributions of shCtrl cells)

**Figure 5:**
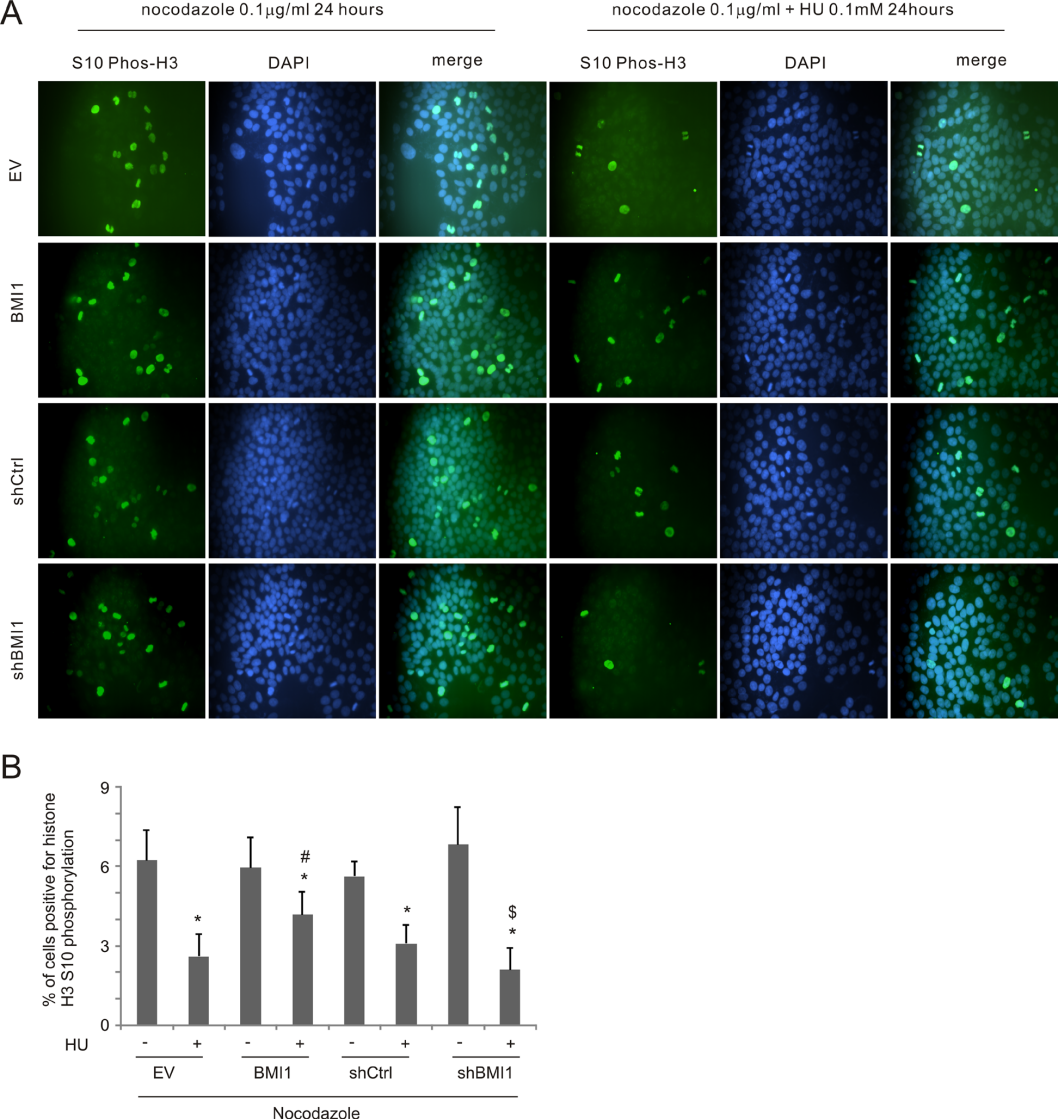
BMI1 decreases HU-elicited S-phase arrest (**A**) MCF7 EV, BMI1, shCtrl, and shBMI1 cells were treated with nocodazole (0.1 μg/ml) without and with HU (0.1 mM) for 24 hours. Histone H3 S10 phosphorylation was determined using IF staining; nuclei were counter-stained with DAPI. Experiments were repeated 3 times; typical images from a single repeat are included (**B**). More than 8,000 nuclei from 12 randomly selected areas per slide were counted to determine the percentage of nuclei positive for histone H3 S10 phosphorylation. Means ± SD (standard deviation) were graphed; *p < 0.05 (two-tailed Student's *t*-test) in comparison to the individual mock treatments, ^#^*p* < 0.05 in comparison to HU-treated EV cells; and ^$^*p* < 0.05 in comparison to HU-treated shCtrl cells.

To further examine BMI1's influence on S-phase arrest in HU-treated cells, MCF7 cells were blocked in mitosis by nocodazole treatment in combination with HU exposure. In these experiments, BMI1-derived reduction of S phase arrest should allow cell cycle progression to mitosis, despite the presence of HU, resulting in mitotic arrest due to the effects of nocodozole. As expected, nocodazole treatment resulted in the accumulation of mitotic cells with condensed chromosomes as detected by immunofluorescence using an antibody to histone H3 phospho-S10 (Figure 5A). HU treatment reduced the number of mitotic nuclei in MCF7 EV cells and this reduction was compromised in MCF7 BMI1 cells, i.e. more mitotic nuclei were observed in HU-treated MCF7 BMI1 cells compared to MCF7 EV cells (Figure 5A, 5B). In contrast, BMI1 knockdown reduced the number of mitotic nuclei in response to HU (Figure 5A, 5B). Collectively, the above observations support the notion that BMI1 reduces S-phase arrest in HU-treated MCF7 cells.

### The involvement of BMI1's structural elements in HU-elicited ATR activation and ATR signalling

**Figure 6:**
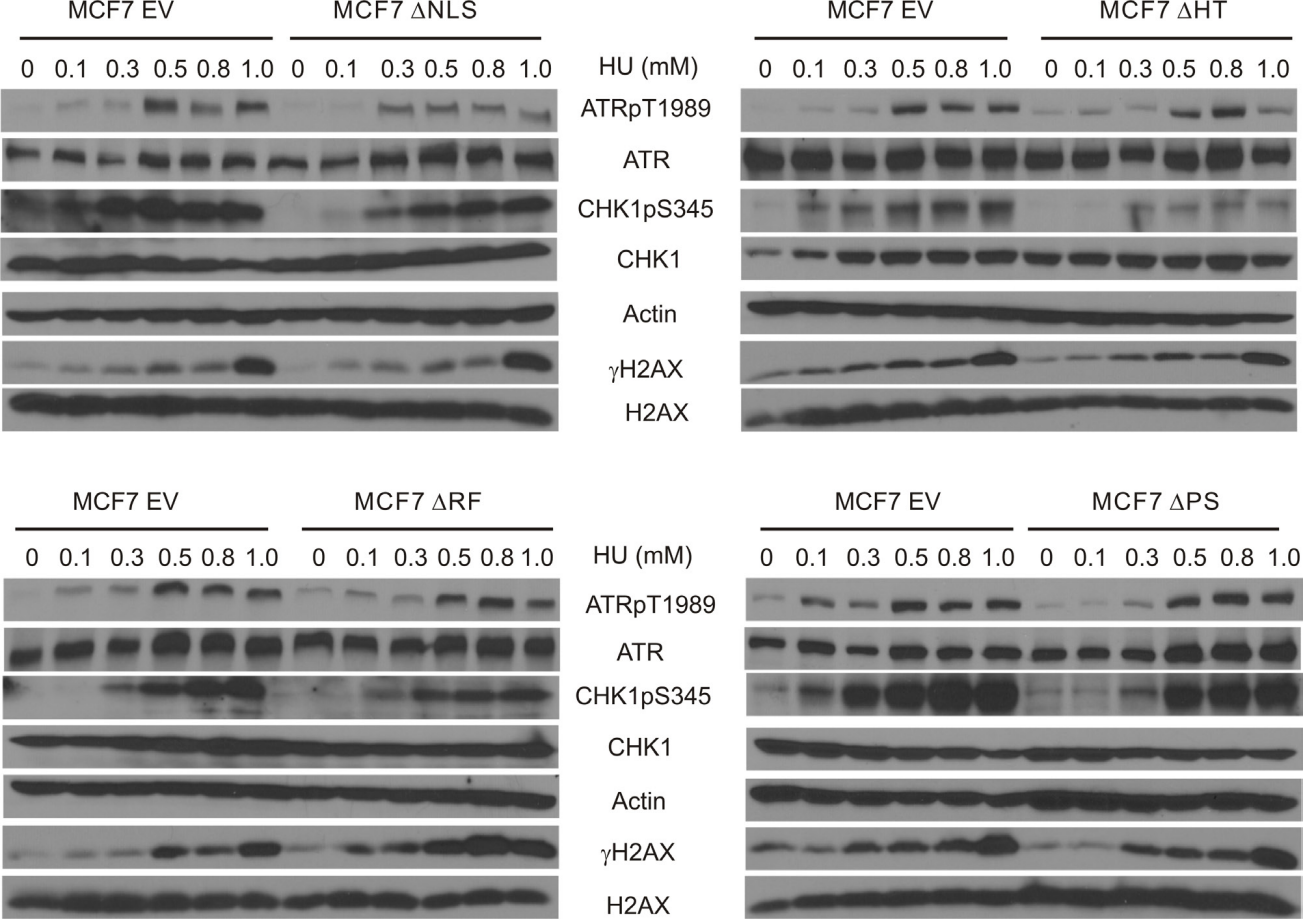
Characterization of BMI1-derived inhibition of ATR activation and ATR signalling MCF7-based EV, BMI1ΔNLS, BMI1ΔHT, BMI1ΔRF, and BMI1ΔPS stable lines have been previously established [31]. These lines were treated as indicated and examined for ATRpT1989, ATR, CHK1pS345, CHK1, actin, γH2AX, and H2AX. Experiments were performed twice. Typical images from a single repeat are shown.

Previously, we have described a set of MCF7 cell lines that stably express individual BMI1 mutants with deletions of the RF, PS, HT, or one of NLS sites [31]. Using these lines, we investigated the generation of ATRpT1989 and two ATR targets (CHK1pS345 and γH2AX) upon HU treatment. While some variation occurs in ATRpT1989 in HU treated MCF7 EV cells, the level of phosphorylation plateaued at 0.5mM HU and remained high at 0.8 and 1mM HU (Figure 6). Within this dose range, all mutants had either a minimal or modest effect on HU-induced ATRpT1989 in comparison to MCF7 EV cells (Figure 6), suggesting that these domains contribute to BMI1-derived inhibition of ATRpT1989. This notion is supported further by the minimal impact of these mutants on HU-induced γH2AX (Figure 6). Nonetheless, all mutants reduced CHK1pS345 in cells treated with HU, although the levels of reductions differed among the mutants (Figure 6). As CHK1pS345 is the best characterized target of ATR [57], these domains may not be essential for BMI1 to reduce ATR signalling (see Discussion for details).

### BMI1 binds TOPBP1

**Figure 7:**
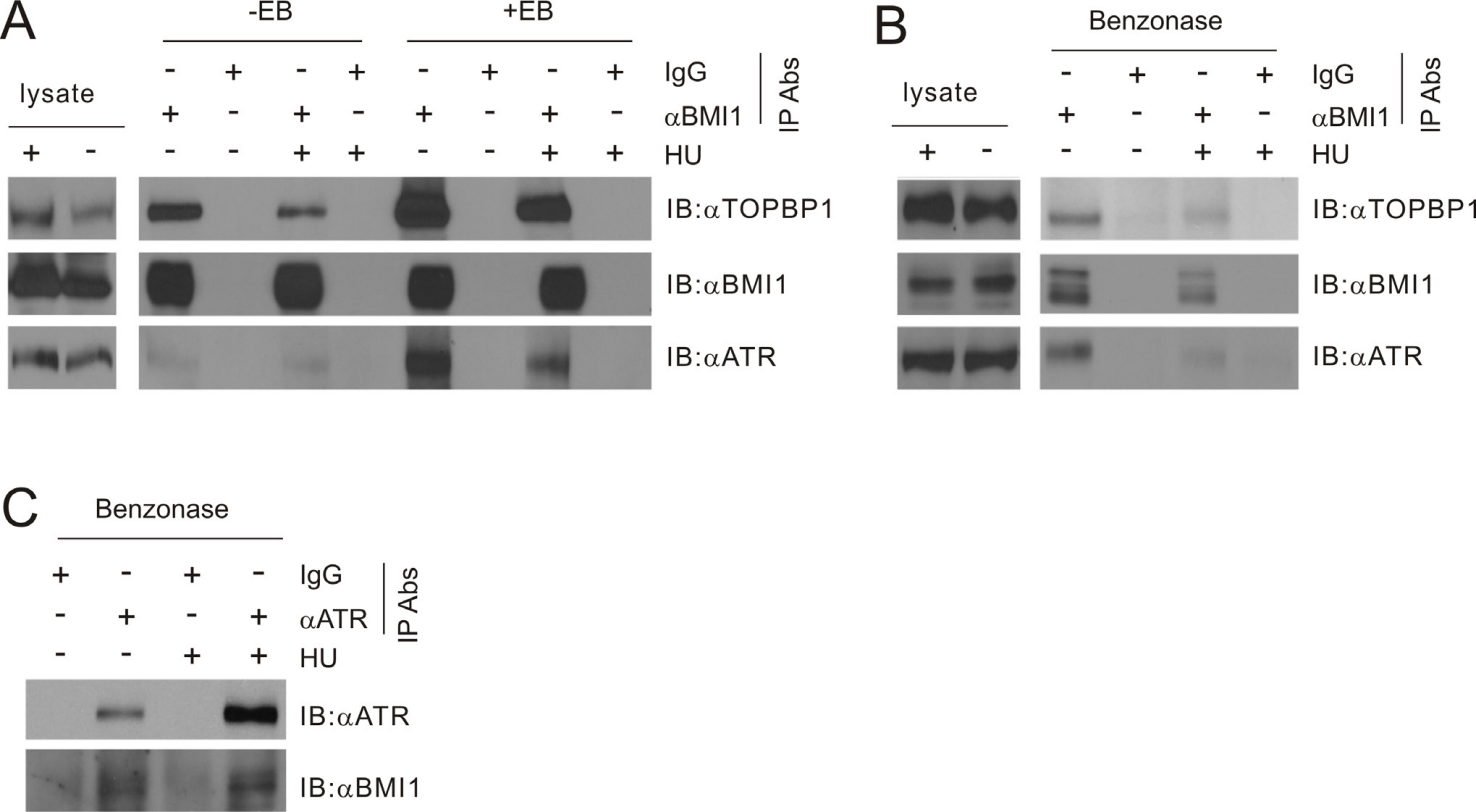
BMI1 associates with TOPBP1 (**A**) 293T cells were transiently co-transfected with BMI1 and TOPBP1 for 36 hours, and treated with HU (1 mM for 24 hours). Cell lysates were then immunoprecipitated (IP) with anti-BMI1 (αBMI1) or control IgG (IgG) in the presence or absence of ethidium bromide (EB, 50 μg/ml; note: cell lysates were pre-incubated with EB for 10 minutes on ice prior to IP) and analyzed by Western blot for TOPBP1, BMI1, and ATR. 1/14 of cell lysates used for IP were also analyzed. (**B, C**) MCF7 cells were treated without or with HU (1 mM for 24 hours). Cell lysates were prepared and treated with benzonase at 1 U/μl on ice for 1 hour, followed by IP for BMI1 (B) or ATR (C), and Western blot examination as indicated. All experiments have been repeated once. Typical images from a single repeat are shown.

ATR activation involves binding to ssDNA and TOPBP1 [41, 58, 59]. The above observations raise the possibility that BMI1 may reduce ATR activation via binding to TOPBP1. In 293T cells co-transfected with BMI1 and TOPBP1, immunoprecipitation (IP) of BMI1 led to co-precipitation of TOPBP1 with and without HU treatment, while HU treatment appeared to reduce the association (Figure 7A, see the ethidium bromide/EB minus lanes). The presence of EB (50 μg/ml) (Figure 7A), a condition that is commonly used to release DNA-associated proteins from DNA [31], did not reduce the efficiency of the co-IP (Figure 7A, compare the respective EB- and EB+ lanes), making it unlikely that the presence of DNA was the cause for the interaction. In fact, the presence of EB apparently enhanced the association, suggesting that interaction between BMI1 and TOPBP1 is possibly mediated by a hydrophobic force, which is enhanced by the presence of positively charged EB.

We further demonstrated the binding of BMI1 and TOPBP1 using the endogenous proteins. In MCF7 cells, immunoprecipitation of BMI1 resulted in co-precipitation of TOPBP1 in the presence of DNase (benzonase endonuclease), further demonstrating that the association was unlikely to be attributable to the presence of DNA (Figure 7B). Similarly, the association between BMI1 and TOPBP1 could be demonstrated with and without HU exposure (Figure 7B). The observed decreases in co-immunoprecipitation of TOPBP1 with BMI1 in HU-treated cells compared to non-treated cells (Figure 7A, 7B) suggest that reductions in this interaction may free TOPBP1 for ATR activation. Collectively, the above observations suggest that BMI1 exists in a protein complex that includes TOPBP1.

### Formation of a potential BMI1/TOPBP1/ATR complex

The binding of BMI1 to TOPBP1 raises the possibility of a BMI1 presence in the TOPBP1/ATR complex. This possibility is supported by the co-IP of endogenous BMI1 through IP of endogenous ATR in the presence of benzonase in MCF7 cells (Figure 7C). The association was observed with and without HU treatment (Figure 7C). Furthermore, the endogenous ATR was co-immunoprecipitated together with the ectopic complex of BM1I-TOPBP1 in 293T cells (Figure 7A) and the endogenous BMI1-TOPBP1 complex in MCF7 cells (Figure 7B). Collectively, this evidence supports the formation of an endogenous BMI1/TOPBP1/ATR complex.

### Characterization of the association of BMI1 and TOPBP1

**Figure 8:**
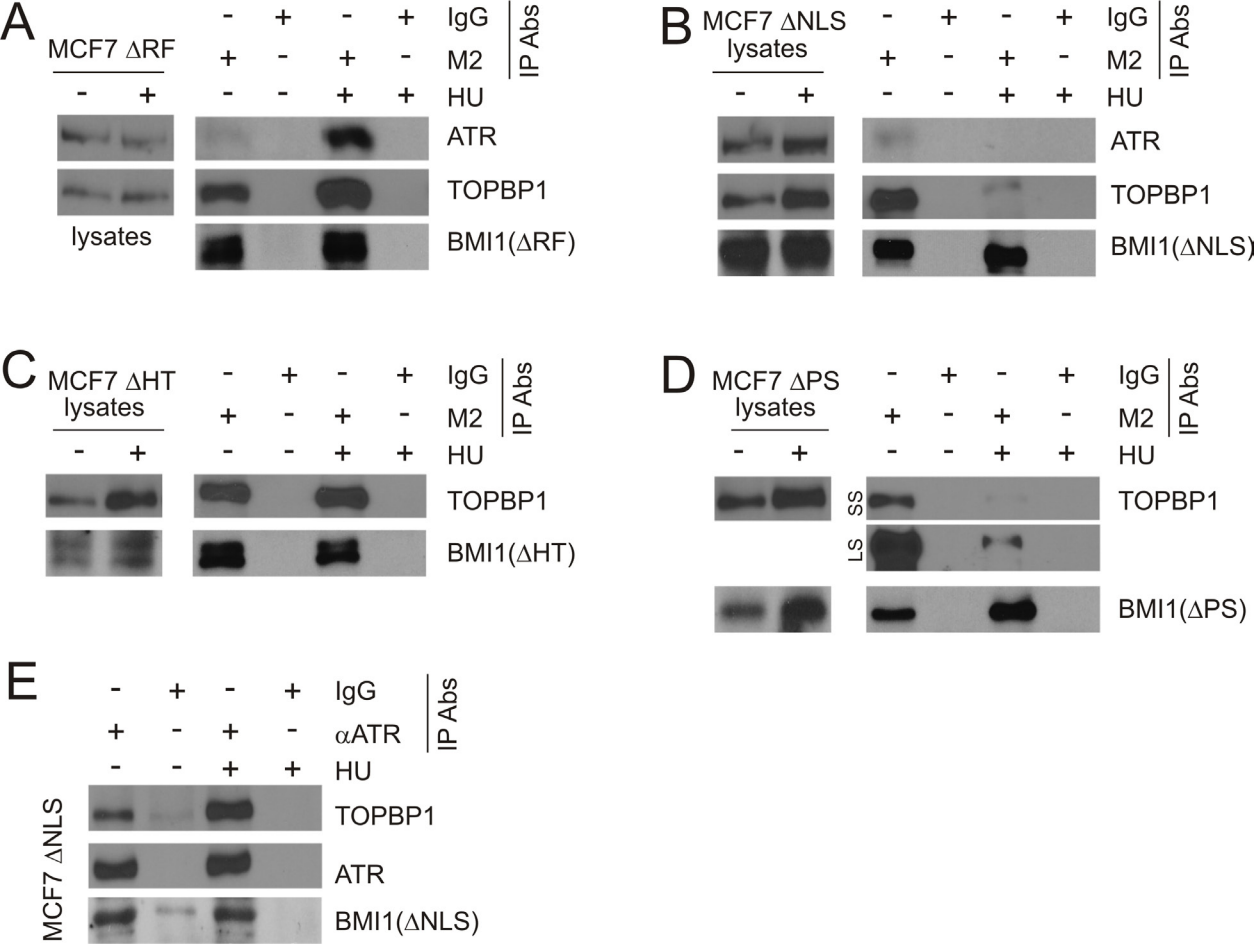
Characterization of BMI1 association with TOPBP1 and ATR Cell lysate from the indicated MCF7 BMI1ΔRF, BMI1ΔNLS, BMI1ΔHT, or BMI1ΔPS cells (**A–E**) were pre-treated with benzonase at 1 U/μl on ice for 1 hour, followed by IP with a monoclonal anti-FLAG (M2) (A–D) or anti-ATR antibodies (E). Western blot analyses were performed for ATR, TOPBP1, and the indicated BMI1 mutants (using a polyclonal anti-FLAG antibody). 1/10 of cell lysates used for IP were also analyzed. Experiments were repeated at least three times; typical results from a single repeat are shown. (D) LS: long exposure; SS: short exposure.

To further study the interaction between BMI1 and TOPBP1, we took advantage of our established MCF7 cell lines stably expressing BMI1ΔRF, BMI1ΔNLS, BMI1ΔHT, or BMI1ΔPS. The expression of the individual BMI1 mutants has been shown in our previous report [31] and has been re-demonstrated here (Figure 8A–8D). As we have reported previously [31], BMI1ΔRF was expressed at a low level in MCF7 BMI1ΔRF cells, which is likely attributable to the mutant protein being unstable. This made detection of the BMI1ΔRF mutant protein difficult in cell lysates [31]. Consistent with our previous report, we observed the mutant in MCF7 BMI1ΔRF cells by real time PCR (data not shown) [31] and IP (Figure 8A) [31]. Importantly, immunoprecipitation of the individual BMI1 mutants through their FLAG tags (using the M2 antibody) resulted in detection of the individual mutant proteins, as expected, along with endogenous TOPBP1 (Figure 8A–8D). With the exception of BMI1ΔRF (Figure 8A), HU treatment either did not affect the association of BMI1ΔHT and TOPBP1 (Figure 8C) or reduced the interaction of TOPBP1 with BMI1ΔNLS (Figure 8B) or BMI1ΔPS (Figure 8D). Collectively, we demonstrated that all BMI1 mutants are capable of interaction with endogenous TOPBP1.

Although we were unable to demonstrate an interaction between ATR and BMI1ΔHT or BMI1ΔPS, we observed co-IP of ATR through BMI1ΔRF or BMI1ΔNLS in cells without exposure to HU (Figure 8A, 8B). Consistent with HU treatment enhancing BMI1ΔRF binding to TOPBP1 (Figure 8A) or reducing TOPBP1 interaction with BMI1ΔNLS (Figure 8B), HU exposure respectively elevated the co-IP of ATR via BMI1ΔRF (Figure 8A) and reduced the co-IP of ATR through BMI1ΔNLS (Figure 8B). Due to a low level of the BMI1ΔRF protein in MCF7 BMI1ΔRF cells (see discussion above for details), the system does not support a reverse co-IP, e.g. co-IP of BMI1ΔRF through ATR. Nonetheless, we have demonstrated co-IP of BMI1ΔNLS through endogenous ATR in MCF7 BMI1ΔNLS cells (Figure 8E). Collectively, these observations further support that BMI1 is able to associate with the TOPBP1/ATR complex.

## DISCUSSION

BMI1 is thought to play an important role in maintaining self-renewal of stem cell populations and it may function in tumorigenesis by repressing tumor suppressor genes through its associated ubiquitin E3 ligase activity. More recent studies have provided evidence of a role for BMI1 in DDR regulation by enhancing HR-mediated DSB repair in mouse embryonic fibroblasts (MEFs), HCT116, and U2OS cells [28, 29]. This function may contribute to resistance towards genotoxic drug-based cancer therapies [29, 60–63]. Additionally, BMI1 may attenuate ATM activation following etoposide-caused DSBs, thereby reducing the duration of the etoposide-initiated G2/M checkpoint [31]. By attenuating DDR signalling, normal levels of BMI1 may favour a return to the cell cycle following DNA lesion repair. In cancer cells with BMI1 upregulation, the elevated levels of BMI1 potentially may compromise DDR, thereby contributing to genome instability, a hallmark of tumorigenesis [33, 34]. Our present results extend these observations by demonstrating that BMI1 can downregulate S-phase checkpoints initiated by ssDNA damage induced by HU in MCF7 and DU145 cells. These observations support a broader role for BMI1 in attenuating DNA damage-induced checkpoint activation.

ATM and ATR are related kinases that share a number of structural features such as the FRAP-ATM-TRRAP (FAT) motif, a kinase domain, and a C-terminal FAT (FATC) domain and presumably they have similar mechanisms of activation [47, 64]. While ATM activation requires the presence of DSBs and binding to NBS1, ATR is activated through association with TOPBP1 on RPA-coated ssDNA [41, 58, 59]. BMI1 attenuates ATM activation through binding to NBS1 [31] and ATR activation through its interaction with TOPBP1 (this study) suggesting that it affects the two kinases through similar mechanisms. In addition, several studies support a role for NBS1 in promoting ATR activation [65–67], raising the possibility that BMI1 can influence both ATM and ATR activation through its interaction with NBS1. However, mutational analysis of BMI1 indicates that BMI1 reduces HU-elicited ATR activation in a different manner from its action in inhibiting etopside-induced ATM activation [31]. Individual deletion of RF, HT, PS or NLS does not compromise BMI1's ability to regulate ATM activation nor the subsequent CHK2 phosphorylation under etoposide-initiated DDR [31], whereas some mutants were largely incapable of inhibiting HU-elicited ATRpT1989. Surprisingly, the mutants are competent in inhibiting ATR-mediated phosphorylation of CHK1 at S345, demonstrating specificity of these domains in reducing ATR activation. These results highlight novel properties of BMI1 in inhibiting ATR signalling. It is possible that BMI1 adopts two different structures or uses different structural elements in inhibiting ATR activation and ATR's ability to phosphorylate CHK1. This possibility is not in contrast to the observations that these mutants are incapable of reducing γH2AX in HU-treated MCF7 cells. A possible explanation is that ATM and DNA-PK also produce γH2AX [46, 68] and DNA-PK can be activated by HU-induced replication stress [69]. Additionally, ATR may phosphorylate S139 of H2AX (γH2AX) in a different manner from phosphorylation of CHK1 at S345. Besides DNA-PK, HU can also activate ATM [70]. ATM and DNA-PK may contribute to CHK1 activation in response to HU treatment; this possibility is consistent with the structural similarities among the three PIKKs: ATM, ATR, and DNA-PK [41, 42]. It is possible that full length and mutant BMI1 proteins reduce HU-induced CHK1 phosphorylation through attenuation of ATM and/or DNA-PK activities. This notion is supported by our experiments demonstrating inhibitory activities of BMI1 towards DSB-induced ATM activation [31]. Nonetheless, despite the above possibilities, ATR is the dominant upstream kinase phosphorylating CHK1 in response to stalled replication forks. In this regard, it is tempting to speculate that BMI1 possesses two properties in reducing ATR activation and decreasing ATR's ability to phosphorylate and activate CHK1.

BMI1 is well studied for its associated E3 ubiquitin ligase activity, a property that requires BMI1's RF domain [2–5]. In the DDR process, BMI1 facilitates DSB repair through the E3 ligase activity [28–30], and reduces ATM activation independently of the enzyme activity [31]. For inhibiting HU-induced ATR activation, the RF motif is required. However, for reducing ATR-mediated CHK1pS345, the RF domain is not essential, which is in accordance with the RF domain being dispensable for BMI1 to associate with either TOPBP1 or ATR. These observations share similarity with the RF domain being non-essential in reducing ATM signaling [31]. It is thus possible that BMI1 reduces ATR activation and signalling in E3 ligase-dependent and -independent processes, respectively. Further research will be required to investigate this issue and the structural elements involved in inhibiting ATR activation and ATR signalling.

BMI1 binds TOPBP1 and ATR. While we cannot distinguish whether BMI1 primarily associates with TOPBP1 or ATR, we favour a model that involves the former. This is because the association of BMI1 with TOPBP1 was consistently detected in comparison to its interaction with ATR. However, this issue should be further investigated. Furthermore, this research is essentially based on enforced expression and knockdown of BMI1. Future research should investigate the impact of endogenous BMI1 on ATR activation and functions.

## MATERIALS AND METHODS

### Materials, cell lines, and cell cycle determination

HU and propidium iodide (PI) were purchased from Sigma Aldrich (Oakville, ON). MCF7 breast cancer and DU145 prostate cancer cell lines were obtained from ATCC, and cultured in DMEM (MCF7) and MEM (DU145) supplemented with 10% FBS (Sigma Aldrich, Oakville, ON) and 1% Penicillin-Streptomycin (Life Technologies, Carlsbad, CA). Cell cycle distribution was examined according to our published procedure [71].

### Immunofluorescence staining

Immunofluorescence staining was performed by fixing cells with prechilled (–20°C) acetone-methanol for 15 minutes, followed by addition of primary antibodies to anti-γH2AX (Cell Signaling, 1:100) or anti-histone H3 S10 phosphorylation (Upstate, 1:250) at 4°C overnight. After rinsing, FITC-Donkey anti-rabbit IgG (1:200, Jackson Immuno Research Lab) was applied for 1 hour at room temperature. Slides were subsequently covered with the DAPI mounting medium (VECTOR Lab Inc.). Images were then acquired with a fluorescent microscope (Carl Zeiss, Axiovert 200).

### Quantification of γH2AX-positive nuclei

More than 200 nuclei from several randomly selected fields of focus were analyzed. Nuclei with ≥ 10 γH2AX foci and those with < 10 γH2AX foci were respectively defined as positive and negative.

### Western blot

Cell lysates were prepared in a buffer consisting of 20 mM Tris (pH 7.4), 150 mM NaCl, 1 mM EDTA, 1 mM EGTA, 1% Triton X-100, 25mM sodium pyrophosphate, 1 mM NaF, 1mM b-glycerophosphate, 0.1 mM sodium orthovanadate, 1mM PMSF, 2mg/ml leupeptin and 10 mg/ml aprotinin. Cell lysates containing 50mg protein were separated on SDS-PAGE gel, and transferred onto Hybond ECL nitrocellulose membranes (Amersham), followed by treatment with 5% skim milk at room temperature for one hour as well as incubation with individual primary and secondary antibodies. Signals were then developed (ECL Western Blotting Kit, Amersham). Primary antibodies used were: monoclonal anti-BMI1 (1:1000, Invitrogen), anti-H2AX (1:1000, Millpore), anti-γH2AX (1:1000, Cell Signaling), anti-T1989 phosphorylated ATR (1:1000, Abcam), anti-ATR (1:500, Santa Cruz), anti- phospho-CHK1 (S345) (1:500, Cell Signaling), anti-CHK1 (1:1000, Cell signaling), anti-TOPBP1 (1:500, Bethyl), anti-phospho-CDK1 (Y15) (1:1000, Cell Signaling), anti-CDK1 (1:1000, Santa Cruz), anti-tubulin (1:1000, Santa Cruz), and anti-actin (1:1000, Santa Cruz).

### Immunoprecipitation (IP)

IP were carried out by incubating 1mg cell lysate proteins with individual antibodies in the presence of Protein G agarose (Invitrogen) or Dynabeads (Invitrogen) overnight at 4°C, and washing 8 times with a buffer [50mM Tris (PH 7.5), 100 mM NaCl, 7.5mM EGTA, and 0.1% Triton X-100]. Lysates were treated with Benzonase (Sigma, 1 U/μl) for one hour on ice. Antibodies used for IP were polyclonal anti-BMI1 (Santa Cruz, 1 μg), anti-M2 (Sigma, 1 μg), and anti-ATR (Santa Cruz, 1 μg). The immunoprecipitations were analyzed by Western blot using anti-BMI1 (1:1000, Invitrogen), anti-TOPBP1 (1:500, Bethyl), polyclonal anti-FLAG (Sigma, 1:500) and anti-ATR (1:500, Santa Cruz).

### Retroviral infection

Retroviral infection was performed according to our published procedure [72]. In brief, a gag-pol expressing vector and an envelope-expressing plasmid (VSV-G) (Stratagene) were co-transfected with a specific retroviral construct into 293T cells for 48 hours. The virus-containing medium was then filtered (0.45 mM filter) and centrifuged (50,000 g for 90 minutes). The viral pellet was resuspended in a specific medium containing 10mg/ml of polybrene (Sigma) prior to infection of cells. Infection was selected using specific antibiotics.

### BMI1 overexpression and knockdown in MCF7 and DU145 cells

MCF7 and DU145 cells were transfected with pBabe and pBabe-based BMI1 retrovirus to establish the respective EV and BMI1 stable cell lines. Control and BMI1 retroviral plasmids expressing short hairpin-based RNAs (shRNA) were purchased from Santa Cruz. Retrovirus was packed and used to generate the respective shCtrl and shBMI1 lines.

### Statistical analysis

Student's *t*-test (2-tails) was used. A *p*-value < 0.05 was regarded as statistically significant.

## SUPPLEMENTARY MATERIALS FIGURES AND TABLES


