# Supplementary material for: BMI1 reduces ATR activation and signalling caused by hydroxyurea
Source: Oncotarget. 2017 Sep 20;8(52):89707–21. doi: 10.18632/oncotarget.21111 (PMC5685703; doi:10.18632/oncotarget.21111)
Supplement: Supplementary file 1 [file oncotarget-08-89707-s001.pdf]

BMI1 reduces ATR activation and signalling caused by hydroxyurea

SUPPLEMENTARY MATERIALS

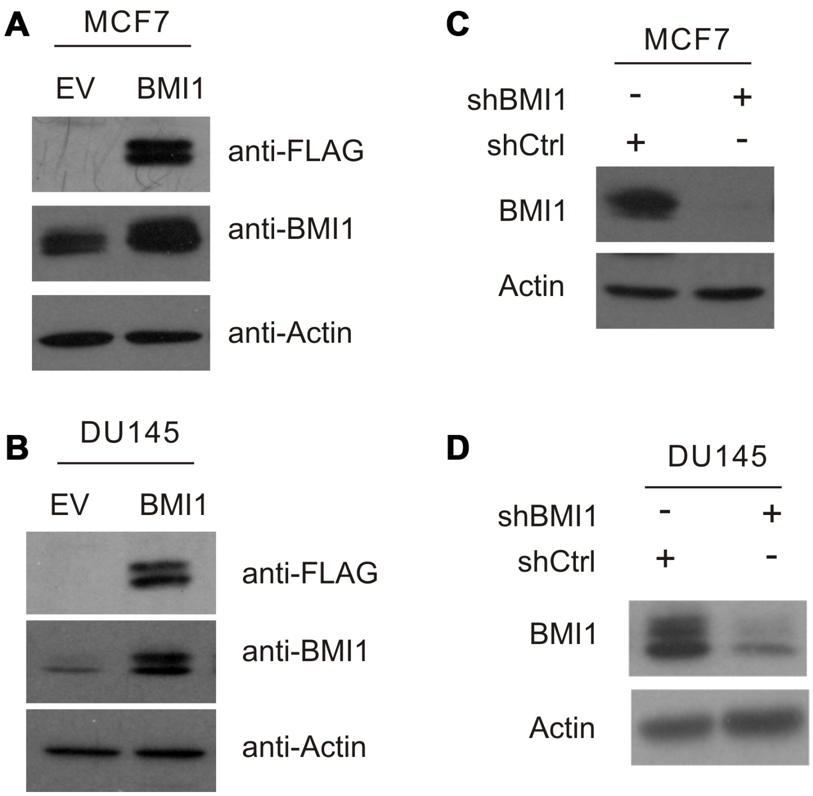

Supplementary Figure 1: Generation of MCF7 and DU145 stable cell lines. (**A**, **B**) Stable expression of FLAG tagged BMI1 in MCF7 and DU145 cell using an empty vector (EV) and BMI1 retrovirus. (**C**, **D**) Stably knockdown of BMI1 using control shRNA (shCtrl) and shBMI1 retrovirus.

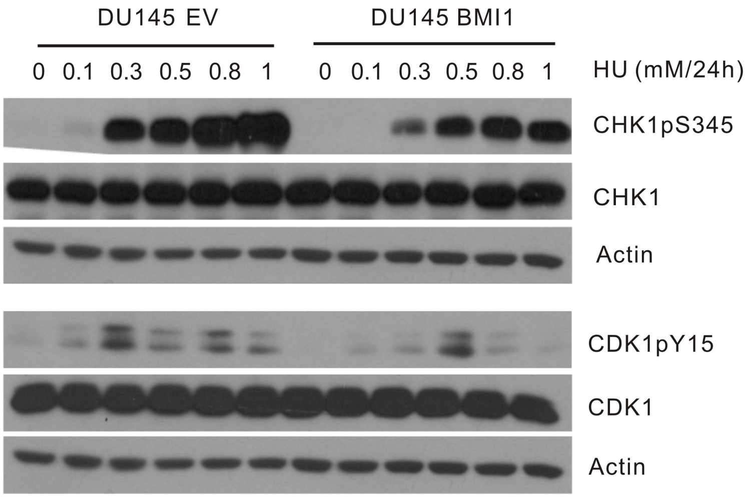

Supplementary Figure 2: Increase in BMI1 reduces CHK1 activation induced by HU. DU145 EV and BMI1 cells were treated with HU as indicated. Western blot analysis was performed to examine phosphorylation of CHK1 at S345 (CHK1pS345), CHK1, phosphorylation of CDK1 at Y15 (CDK1pY15), CDK1, and actin. Experiments were performed twice; typical results from a single repeat are included.

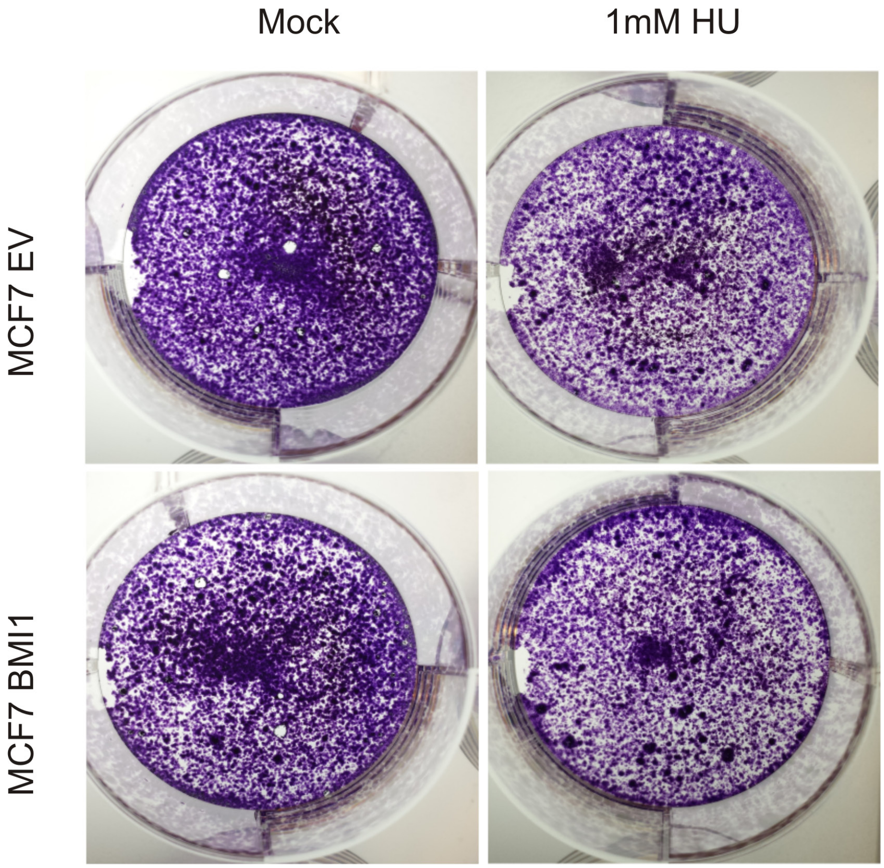

Supplementary Figure 3: MCF7 EV or BMI1 cells renew proliferation after HU treatment. The indicated MCF7 cells (104) were mock-treated (PBS) or treated with 1mM HU for 24 hours. HU was then removed; cells were rinsed and cultured in normal medium for 9 days. Surviving cells were stained with 0.5% crystal violet. Experiments were repeated once. Typical images from a single repeat are shown.

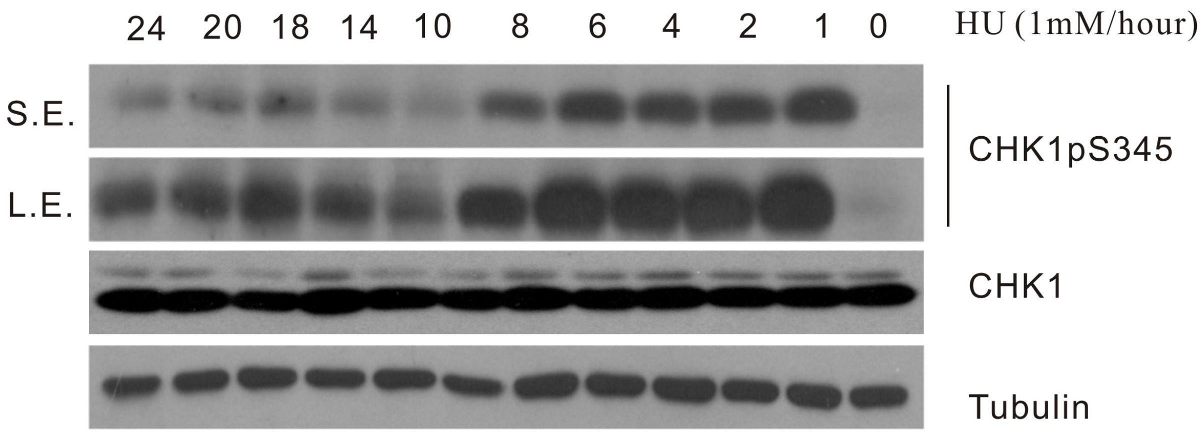

Supplementary Figure 4: Kinetics of HU-induced CHK1pS345. MCF7 cells were treated as indicated. Western blot analysis was then performed to examine CHK1pS345, CHK1, and tubulin. The CHK1pS345 signals were obtained by a short exposure (S.E.) and long exposure (L.E.).

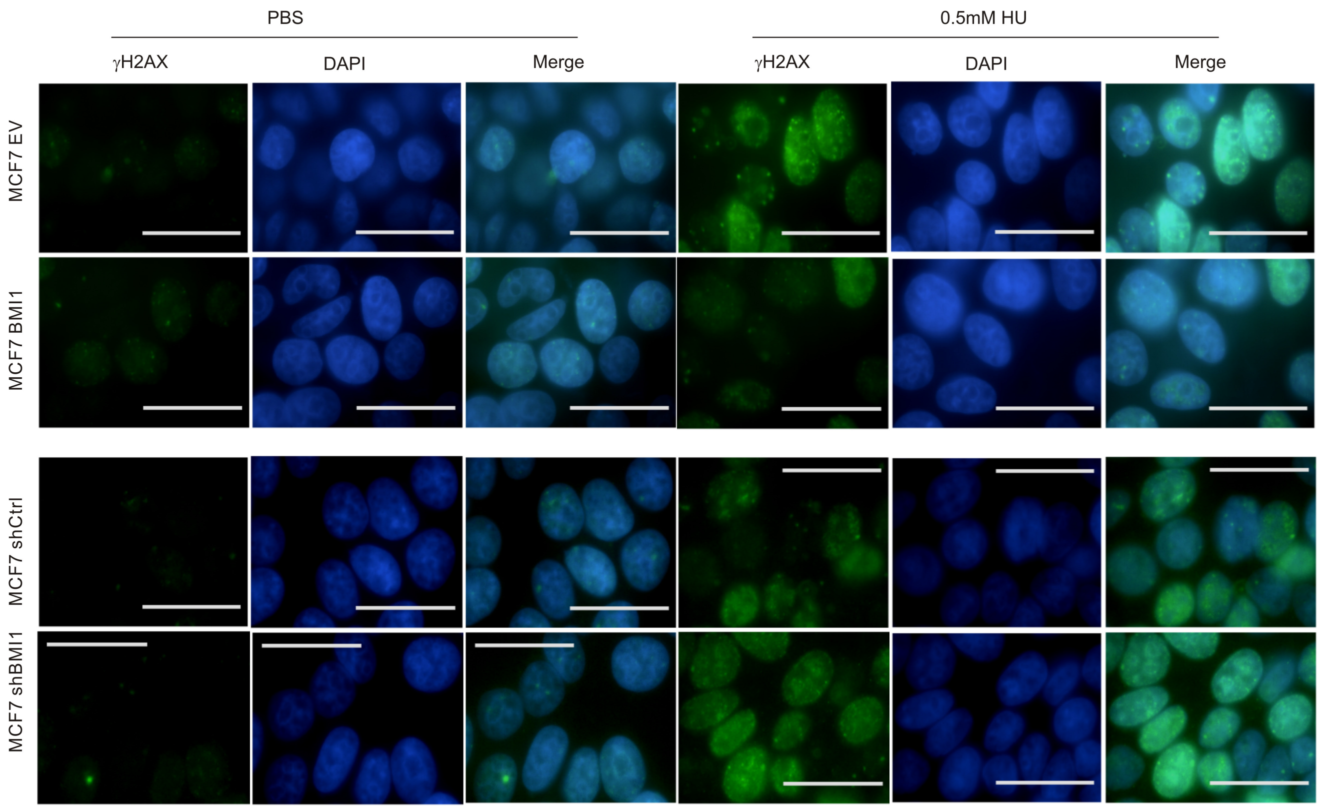

Supplementary Figure 5: BMI1 attenuates gH2AX in cells treated with HU. MCF7 EV, BMI1, shCtrl, and shBMI1 cells were treated with PBS (control) or HU for 24 hours. IF staining for gH2AX was performed; nuclei were counter stained with DAPI (blue). Scale bars are for 20 µm. Experiments were repeated three times; typical images from a single repeat are shown.

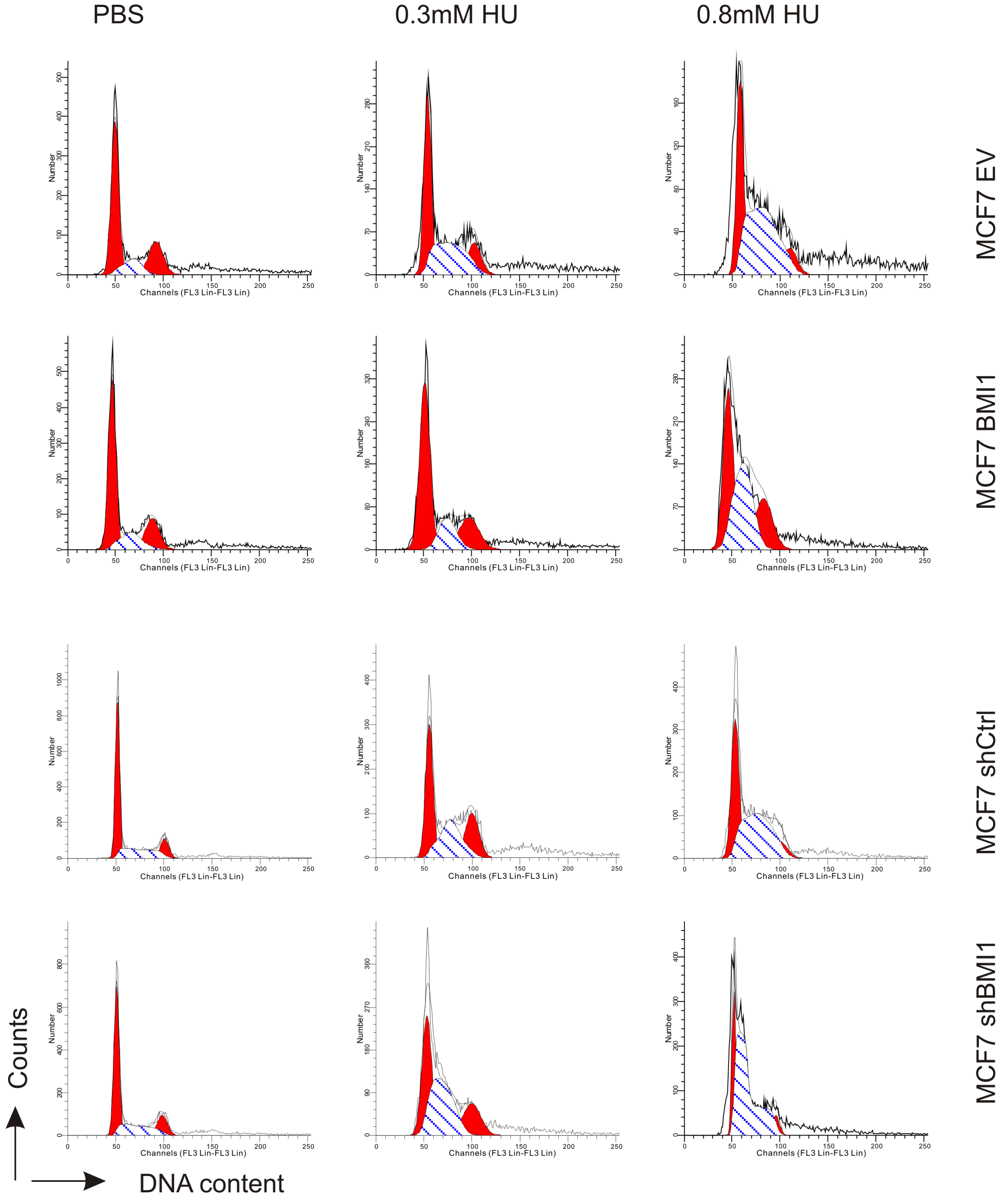

Supplementary Figure 6: BMI1 decreases HU-elicited S-phase arrest. MCF7 EV, BMI1, shCtrl, and shBMI1 cells were treated with PBS, 0.3 mM HU, or 0.8 mM HU for 24 hours. Cell cycle distribution was determined using a flow cytometer.
